# Effectiveness of Ozone Therapy in Non-Surgical Periodontal Treatment: A Meta-Analysis of Topical Applications

**DOI:** 10.3390/jcm14145124

**Published:** 2025-07-18

**Authors:** Alessia Pardo, Annarita Signoriello, Gabriele Brancato, Raffaele Brancato, Elena Messina, Paolo Faccioni, Stefano Marcoccia, Gianna Maria Nardi, Giorgio Lombardo

**Affiliations:** 1 Dentistry and Maxillo-Facial Surgery Unit, Department of Surgery, Dentistry, Pediatrics and Gynecology (DIPSCOMI), University of Verona, Piazzale L.A. Scuro 10, 37134 Verona, Italy; alessia.pardo@univr.it (A.P.); annarita.signoriello@univr.it (A.S.); gabriele.brancato.98@gmail.com (G.B.); raffa7654@gmail.com (R.B.); giorgio.lombardo@univr.it (G.L.); 2 Dentistry Unit, Department of Surgery, Mater Salutis Hospital, Via Gianella 1, 37045 Legnago, Italy; stefano.marcoccia@univr.it; 3 Department of Odontostomatological and Maxillofacial Sciences, “Sapienza” University of Rome, 00185 Roma, Italy; giannamaria.nardi@uniroma.it

**Keywords:** non-surgical periodontal therapy, ozone therapy, periodontitis

## Abstract

**Background:** Additional therapies (e.g., laser, photodynamic therapy, and ozone) have been reported to improve mechanical instrumentation and immune response in non-surgical periodontal therapy (NSPT). With this systematic review we evaluated the effectiveness of ozone therapy in reducing inflammation and progression of periodontal disease. **Methods:** Three electronic databases (PubMed, Scopus, and Cochrane Library) were searched for randomized and clinical trials on ozone therapy (gas, liquid, gel/oil) combined with NSPT. The study design followed Preferred Reporting Items for Systematic Reviews and Meta-Analyses (PRISMA) 2020 guidelines and the risk of bias was assessed using the RoB-2 tool. **Results:** Eight of the twenty-two studies reviewed reported on gaseous ozone, nine on ozone water, and five on ozonated oil/gel as an adjunct to mechanical periodontal instrumentation, often with scaling and root planing (SRP). Ozone was found to be more effective than SRP alone in treating inflammation, as measured with the gingival index (VMD −0.32; 95% confidence interval (CI) (−0.41; −0.24); *p* < 0.00001) and compared to chlorhexidine (CHX) (ozone gel; VMD −0.10; 95% CI (−0.20; −0.01); *p* = 0.03). The study findings were inconsistent, however, with several reporting clinical and microbiological benefit while others observed no marked improvement with the addition of ozone therapy to NSPT. **Conclusions:** While ozone therapy may represent a useful adjunct to NSPT, further research with larger study groups is warranted to determine its effectiveness.

## 1. Introduction

Over the past three decades the incidence of periodontitis has increased and is now reported as the twelfth most prevalent pathology worldwide [1]. A hallmark of this chronic multifactorial disease is progressive inflammation and destruction of the supporting dental structures, leading to spontaneous or probing-induced bleeding (BOP), clinical attachment level (CAL), increased probing pocket depth (PPD), and radiographic marginal bone loss (MBL) [2]. The progression of inflammation in periodontitis is linked to the persistence of periodontal pockets, which create a suitable environment for the proliferation of anaerobic pathogenic microorganisms [3]. Anaerobic bacteria are the primary cause of bone loss, with more than 500 different species capable of colonizing the oral cavity in adults [4,5] and promoting disease progression from initial onset to severe stages of periodontal tissue destruction [3].

Mechanical therapy, combined with patient education and rigorous compliance, is considered an optimal evidenced-based treatment to reduce inflammation caused by bleeding and interrupt the progression of disease [6]. Scaling and root planing (SRP) is widely accepted as the gold standard for non-surgical periodontal treatment (NSPT) to remove supra- and subgingival dental plaque and calculus and to smooth the root surfaces infected by bacteria [7,8,9].

This procedure is performed using manual instruments (such as curettes and scalers) and mechanical devices, including sonic, piezoelectric, and magnetostrictive scalers. The therapeutic goal of SRP is to halt the progression of periodontal disease by eliminating subgingival biofilms and reducing periodontal pocket depth, thereby preventing further loss of clinical attachment and ultimately avoiding tooth loss [6].

Despite its fundamental role in periodontal therapy, SRP has clinical limitations. A residual periodontal pocket depth (PPD) of 6 mm or more after treatment is considered an incomplete result, as SRP does not completely eliminate periodontal pathogens, particularly in deep or anatomically complex pockets. These sites often have restricted access points and complex root morphologies, which compromise the effectiveness and efficiency of subgingival instrumentation [9].

SRP alone may not always be sufficient, however. Studies [10,11,12,13,14] have shown that adjunctive therapy for periodontitis (e.g., laser, photodynamic therapy, or ozone) can improve immunogenic response and outcome after NSPT.

Moreover, local treatments [10,11,12,13] should be preferred over systemic treatment, such as antibiotics [14] which are often associated with the risk of developing bacterial resistance [15].

Ozone is a natural triatomic gas known for its protective role against ultraviolet radiation and its remarkable medicinal properties [16]. Found in the stratosphere at concentrations of 1–10 parts per million (ppm), it is continuously created and degraded into molecular oxygen [17], thus playing a vital role in protecting life on Earth [18]. At room temperature, ozone is a colorless gas with a strong odor; it is detectable at concentrations as low as 0.02–0.05 ppm [19]. Owing to its bactericidal, virucidal, fungicidal, immune-modulatory, and anti-inflammatory properties, ozone is employed in various applications for dental treatment [16], for instance, to manage wound healing, mandibular osteonecrosis, post-surgical pain, dental caries, root canal treatment, dentin hypersensitivity and teeth whitening, temporomandibular joint disorders, oral lichen planus, gingivitis, periodontitis, and halitosis, and, in particular, for the efficient elimination of bacterial plaque and biofilm [20].

In periodontology, ozone can be applied in various forms, including gaseous ozone, ozonated water, and ozonated olive or sunflower seed oils/gels.

These can be used as pre- and post-therapy mouthwashes or administered directly into periodontal pockets. Ozone exhibits potent antimicrobial and anti-inflammatory properties, attributed to its ability to oxidize microbial cell walls and modulate host immune responses [17]. The effectiveness of ozone therapy depends on factors such as the form of ozone administration, the concentration used, the duration of application and the specific periodontal condition to be treated.

As many of the bacteria responsible for periodontal disease are anaerobic, the release of oxidants with the use of ozone can reduce bacterial growth by increasing oxygen tension in the subgingival biofilm and by damaging intracellular components. Previous studies [21,22] have observed that ozone demonstrates excellent biocompatibility with gingival fibroblasts and that it stimulates host metabolism and immune-cell activity [20,21]. For this reason, ozone is currently used as antibacterial agent in healthcare and dental practice, with its well-established application as gas, water, or gel/oil [10].

The concentrations commonly used for ozone therapy vary according to the formulation: gaseous ozone is typically applied at 10–40 mg/L, ozonated water at approximately 0.5 mg/L, while the concentration of ozone in gel formulations is generally specified by the manufacturer. These values may vary depending on the type of ozone-generating equipment and the specific product used. In light of the above [15,16,17,18,19,22], we evaluated the effectiveness of ozone therapy (gaseous ozone, ozonated water, and ozonated oil) as an adjunct to NSPT in reducing BOP and inflammation (gingival index, GI). The secondary aim of this review was to analyze its effectiveness in reducing probing pocket depth (PPD) and clinical attachment level (CAL).

According to the null hypothesis, there is no significant difference in the variation of BOP (bleeding on probing) and GI (gingival index) between the group treated with ozone therapy in addition to non-surgical periodontal therapy (NSPT) and the group treated with NSPT alone.

## 2. Materials and Methods

This systematic review and meta-analysis were conducted in accordance with the Preferred Reporting Items for Systematic Review and Meta-Analyses [23] and registered with PROSPERO under number CRD42025630901.

### 2.1. Inclusion and Exclusion Criteria

The inclusion and exclusion criteria are presented in Table 1.

The PICO (population, intervention, comparison, outcome) outline was as follows:

− P (population): patients diagnosed with periodontal disease;
− I (intervention): non-surgical periodontal therapy + ozone gas, liquid, and gel/oil therapy;
− C (comparison): non-surgical periodontal therapy + placebo or another adjunctive therapy without any form of ozone;
− O (outcome): main clinical parameters of bleeding on probing (BOP) and gingival index (GI), and secondary parameters of probing pocket depth (PPD) and clinical attachment level (CAL).

### 2.2. Information Sources and Search Strategy

Three electronic databases (PubMed, Scopus, and Cochrane Library) were searched for randomized clinical trials (RCT) and clinical trials published between 2009 and 2025 on ozone therapy (gaseous, liquid, or gelatinous/oily formulation) and NSPT using the search terms: “periodontal disease OR periodontitis OR periodontics OR dental AND ozone water OR ozonated water OR ozone treatment OR ozone oil OR ozonated oil OR ozone OR ozone gel OR gaseous ozone OR ozonide”. Since two of the filters could not be applied to the search string in the Cochrane Library, the search terms “Randomized Controlled Trials” OR “Clinical Trials” were added.

### 2.3. Screening, Selection, and Data Collection/Extraction

Two reviewers (RB and AP) independently selected studies for review and meta-analysis by evaluating titles, abstracts, and full texts of RCTs or clinical trials. Uncertainties regarding study eligibility were resolved either between the reviewers or with the assistance of a third reviewer (GB). To ensure consistency in study selection and enhance reliability of the search strategy and data selection/collection/extraction, a subset of studies was selected for calibration (15% of the total retrieved) and independent analysis by all three reviewers.

Inter-rater reliability between the reviewers was calculated with Cohen’s kappa coefficient and the process repeated until acceptable agreement was achieved (between 0.6 and 0.8). Calibration was reported, including initial and final agreement levels, and discussion discrepancies, while refining inclusion criteria as necessary and reassessing agreement during the full review process.

### 2.4. Risk of Bias

Two reviewers (RB and AP) assessed the risk of bias. The RoB-2 tool for RCTs was used for the analysis [24]. The tool is composed of questions in five domains (randomization process, deviations from the intended interventions, missing outcome data, measurement of the outcome, and selection of the reported result), to reach a final score categorized as “low risk”, “some concerns”, or “high risk”. In the present review, if all domains were assessed as “low risk”, the study was assigned a “low risk of bias”. If at least one domain showed “some concerns” and none had a high risk, the study was rated as “having some concerns”. If at least two domains showed “some concern” or one domain showed “high risk”, the study was rated as “having a high risk of bias”.

### 2.5. Effect Measures

In the meta-analysis, continuous outcomes were assessed by calculating the number of samples, means, and standard deviations for each outcome. Statistical analysis was performed on studies with comparable outcomes, comparing the ozone treatment group (all formulations) with the control group for four clinical parameters (BOP, GI, CAL, and PPD). Additionally, an analysis compared the ozone gas and the ozonated water treatment groups versus the control group. Finally, an analysis of PPD and the GI compared the ozone gel with the CHX treatment group.

Review Manager Web software (Cochrane Collaboration) was used to analyze the quantitative data [25]. Meta-analysis was performed using a fixed-effects model, inverse-variance method, average differences, or standardized average differences according to the studies and parameters. A 95% confidence interval (CI) was calculated for the primary results. Forest plots were then generated and heterogeneity and overall effect tests were conducted. Sub-group analysis was used to explore the causes of statistical heterogeneity and differentiate studies involving different age groups. The I^2^ index was used to quantify statistical heterogeneity.

### 2.6. Certainty Assessment

Two reviewers (RB and AP) used the GRADEpro Guideline Development Tool (GDT) to assess confidence in the evidence [26] according to risk of bias, inconsistency, indirectness, imprecision, publication bias, large effect, plausible confounding, and dose–response gradient. In instances of disagreement, the reviewers discussed ways to reach consensus on confidence assessment.

## 3. Results

### 3.1. Study Selection

**Figure 1:**
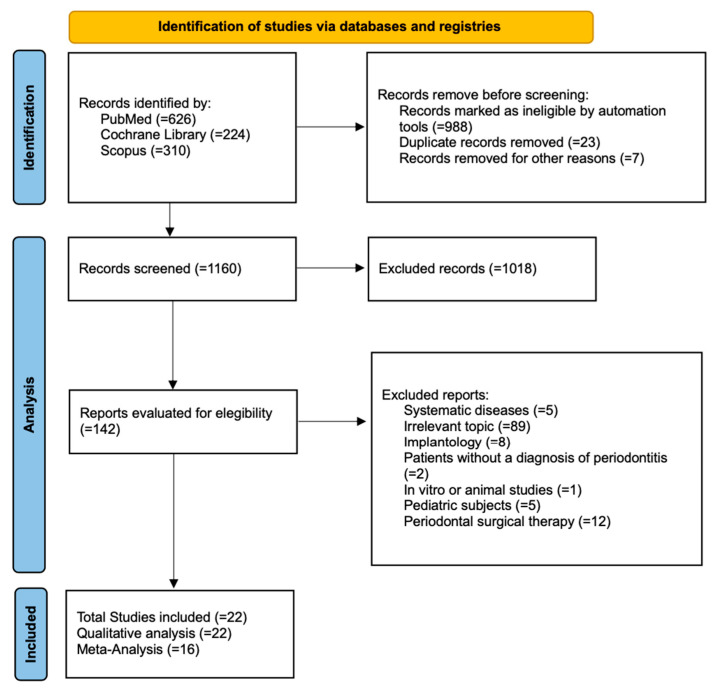
Study flowchart based on PRISMA 2020 guidelines [23].

A total of 1160 studies were identified by the database search: 626 from PubMed, 224 from the Cochrane Library, and 310 from Scopus. Before screening, 1018 studies were excluded as they were found to be ineligible by the automation tool (n = 988), because they were duplicates (n = 23), or for other reasons (n = 7). A total of 142 studies were then analyzed, 122 of which were excluded because they dealt with systemic diseases (n = 5), were irrelevant to the topic (n = 89), dealt with implantology (n = 8), did not report a diagnosis of periodontitis (n = 2), were in vitro or animal studies (n = 1), involved children (n = 5), or reported periodontal surgical therapy (n = 12). Twenty-two studies, all RCTs, met the inclusion criteria and were reviewed. Figure 1 presents the study flow chart.

### 3.2. Study Characteristics

A total of 22 studies underwent qualitative analysis and are described below according to the type of ozone therapy used (gaseous ozone, ozonated water, or ozonated oil/gel).

#### 3.2.1. Gaseous Ozone

Eight studies were RCTs published between 2010 and 2022 (Table 2): six had a split-mouth and two had a full-mouth design. The number of patients varied from study to study (maximum 90 subjects) [26]. The age range also varied (average age 40 years). All involved scaling and root planing (SRP) and gaseous ozone as the test interventions and were compared with laser therapy [27], placebo [27,28], air [29], or CHX [26,27,28,29,30] (Table 1). All studies measured plaque index (PI), gingival index (GI), clinical attachment level (CAL), and bleeding on probing (BOP). Alongside clinical parameters, some studies also evaluated specific biomarkers, including 8-OHdG (8-hydroxyguanosine), MDA (malondialdehyde), TGF-β (transforming growth factor beta) oxidative stress and inflammation markers [28], IL-1β (interleukin-1), Hs-CRP (high-sensitivity C-reactive protein) inflammatory markers [31], and microorganisms, which were qualitatively analyzed in one study [29]. The follow-up varied across studies: six had a short-term follow-up (1 week, 1 month, 3 months) [27,28,29,30,31,32] and two had a 6-month follow-up [26,33].

#### 3.2.2. Ozonated Water

Most of these nine RCTs published between 2010 and 2023 had a split-mouth design [34,35,36,37,38,39], while the others had a full-mouth design [40,41,42]. All used ozonated water, with one study specifically using nanobubble ozone water (NBW3) [41] (Table 3).

The control group received distilled water (placebo) [41,42], saline irrigation [36], photodynamic therapy (PDT) [35], or CHX gel 0.20% [37,38]. Common clinical indicators were PI, GI, PPD, CAL, and BOP. Some studies included microbiological analysis [35,38,40,41,42], with specific attention paid to the red complex of pathogenic bacteria [41]. Some assessed inflammatory biomarkers (IL-1β and CRP) [34,36]; the age range was 30 to 65 years. The follow-up was 2 [35,36,38,39,40,41,42] to 3 months [34,37].

#### 3.2.3. Ozonated Olive Oil/Gel

Five RCTS, published between 2012 and 2022, had a split-mouth design [14,43,44,45,46] (Table 4). Four investigated ozonated olive oil [14,44,45,46], while one used ozonated gel and ozonated sunflower seed oil [43]. The sample size ranged from 10 to 30 subjects. The most common control treatment was CHX gel 1% [14,43,44,46], with one study using CHX gel 0.20% [45]. Common clinical indicators were PPD, CAL, PI, GI, and BOP. One study included microbiological analysis [45] and another incorporated subjective parameters, such as the visual analog scale (VAS) for pain [44]. The follow-up was up to 3 months in four studies [14,44,45,46] and up to 6 months in one study [43].

### 3.3. Microbiological Outcomes

#### 3.3.1. Gaseous Ozone

Butera et al. [33] reported a reduction in pathological sites after 6 months (T3) from 84.11% to 40.88% in the Ozone + SRP group and from 74.54% to 63.04% in the SRP group alone. They found a marked improvement in the ozone-treated group, with a decrease in almost half of pathological sites. Ramirez Peña et al. [29] reported that gaseous ozone significantly (*p* < 0.001) reduced anaerobic colonization (moderate-risk colonies of cocci and bacilli, and high-risk colonies with spirochetes) at 3 weeks. Uraz et al. [30] found that SRP and SRP + ozone significantly reduced bacterial count at 1 month (*p* < 0.001); *Phorphyromonas gingivalis* at 1 month (*p* = 0.006); *Tannerella forsythia* (*p* = 0.004); and *Prevotella intermedia* (*p* = 0.01) at 3 months. No significant difference in reduction between the two treatments was reported.

#### 3.3.2. Ozonated Water

Shrivastava et al. [41] and Vasthavi et al. [42] reported no significant changes in microbiological data between the test and the control groups. Mehrotra et al. [35] found a significant reduction in pathogens in both groups: 53.8% in the SRP + ozone water group (*p* < 0.001) and 69.2% in the SRP + photodynamic therapy group (*p* < 0.001) at 2 months. There was no statistical difference between the groups (*p* = 0.2). Hayakumo et al. [40] reported no significant overall reduction in the number and the mean percentage of *P. gingivalis* and *T. forsythia* at 8 weeks (*p* > 0.05): there was a ≥95% reduction in *P. gingivalis* in the test group and a 50% reduction in the control group at 8 weeks; there was a ≥95% reduction in *T. forsythia* in the two groups. Kshitish et al. [38] found a 50% reduction in *Aggregatibacter actinomycetemcomitans* after ozone therapy but no change in *P. gingivalis* or *T. forsythia*. The control treatment (CHX) had no effect on *A. actinomycetemcomitans*, *P. gingivalis*, or *T. forsythia.* Unlike treatment with CHX, ozone therapy reduced *Candida albicans* to almost nil after 1 min.

#### 3.3.3. Ozonated Oil/Gel

Gandhi et al. [45] reported no significant difference between ozone gel and CHX (control group). Patel et al. [44] found that ozone therapy combined with SRP can be significantly (*p* < 0.05) more effective than SRP alone.

### 3.4. Risk of Bias Evaluation

**Figure 2:**
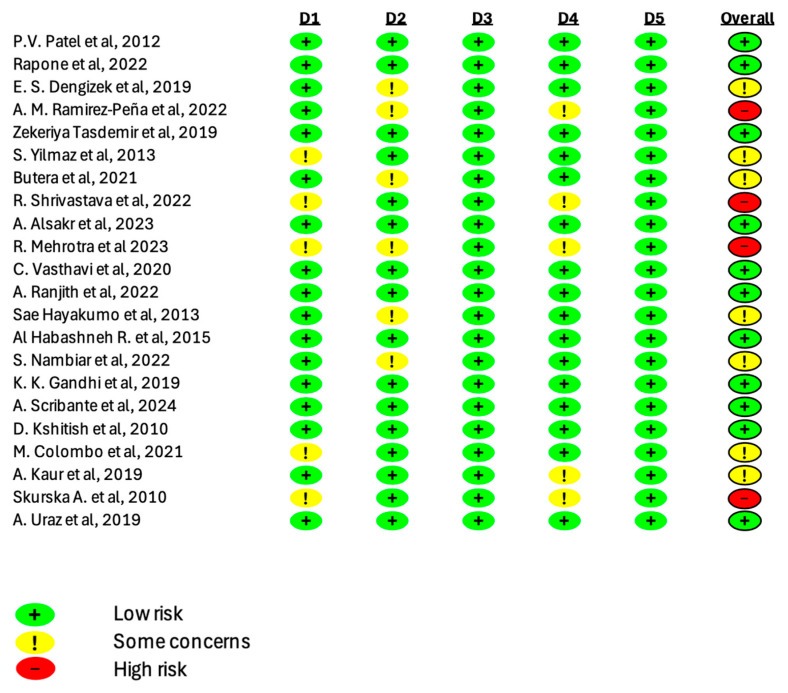
Assessment of the risk of bias in Rob-2 randomized clinical trials [14,24,26,27,28,29,30,31,32,33,34,35,36,37,38,39,40,41,42,43,44,45,46].

Figure 2 presents the results of the Rob-2 tool to assess the risk of bias in RCTs. The overall risk ranged from “low” to “some concerns”; the overall risk in four RCTs was “high”.

### 3.5. Meta-Analysis

**Figure 3:**
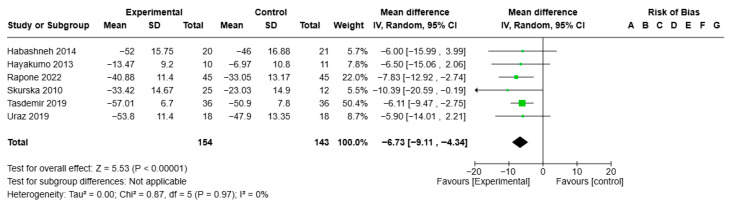
Forest plot of the meta-analysis of BOP [26,30,31,32,34,40].

A preliminary statistical analysis of BOP for the ozone treatment group (all forms) versus the control group showed VMD −6.73, 95% CI (−9.11; −4.34), I2 of 0%; the test for overall effect was statistically significant (*p* < 0.00001) (Figure 3).

**Figure 4:**
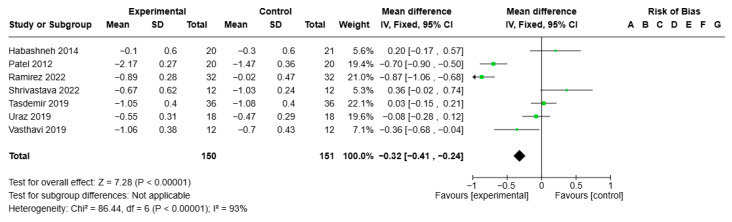
Forest plot of the meta-analysis of the GI [29,30,31,34,41,42,44].

Statistical analysis of the GI for the ozone treatment group (all forms) versus the control group showed VMD −0.32, 95% CI (−0.41; −0.24), I2 of 93%, and statistical significance (*p* < 0.00001) (Figure 4).

**Figure 5:**
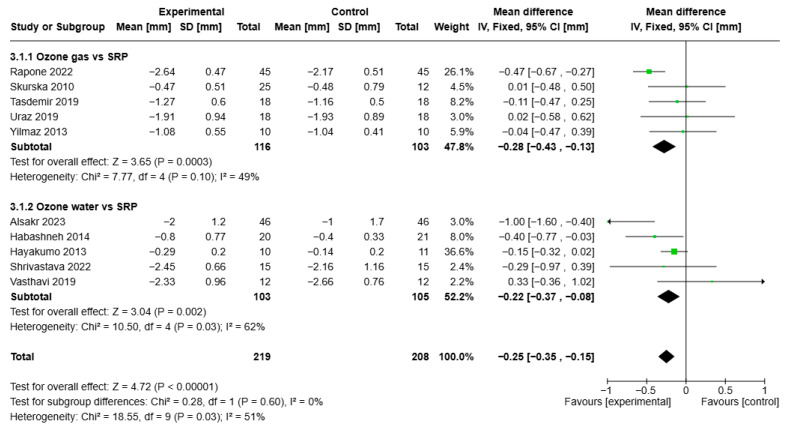
Forest plot of the meta-analysis of PPD (ozone gas and ozone water vs. SRP) [26,27,30,31,32,34,39,40,41,42].

Statistical analysis of PPD for the ozone treatment group (all formulations) versus the control group showed VMD −0.25, 95% CI (−0.35; −0.15), I2 of 51%, and statistical significance (*p* < 0.00001). The first group analysis of ozone gas showed VMD of −0.28, 95% CI (−0.43; −0.13) with I2 of 49% and statistical significance (*p* < 0.0003). The second group receiving ozone water showed VMD −0.22, 95% CI (−0.357; −0.08), I2 of 62%, and statistical significance (*p* = 0.002) (Figure 5).

**Figure 6:**
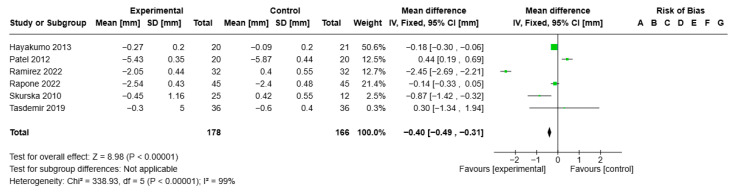
Forest plot of the meta-analysis of CAL [26,29,31,32,40,44].

Statistical analysis of CAL for the ozone treatment group (all formulations) versus the control group showed VMD −0.40, 95% CI (−0.49; −0.31), I2 of 99%, and statistical significance (*p* < 0.00001) (Figure 6).

**Figure 7:**
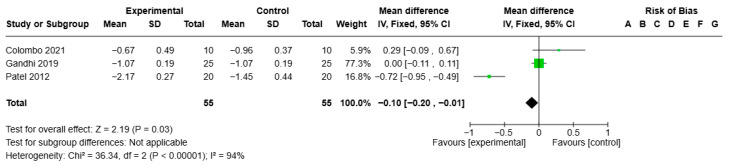
Forest plot of the meta-analysis of the GI (ozone gel vs. CHX) [14,44,45].

Statistical analysis of the GI for ozone gel treatment versus CHX showed VMD −0.10, 95% CI (−0.20; −0.01), I2 of 94%, and statistical significance (*p* = 0.03) (see Figure 7).

**Figure 8:**
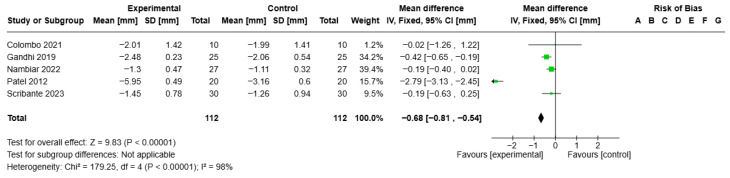
Forest plot of the meta-analysis of PPD (ozone gel vs. CHX) [14,43,44,45,46].

Statistical analysis of the PPD for the ozone gel treatment versus CHX treatment showed VMD −0.68, 95% CI (−0.81; −0.54), I2 of 98%, and statistical significance (*p* = 0.00001) (see Figure 8).

### 3.6. Grading the Body of Evidence

The evidence and the strength of recommendations were evaluated according to GRADEpro GDT. Moderate certainty indicates we can be moderately confident in the effect estimate (Table 5 and Table 6).

## 4. Discussion

Periodontitis is a public health issue and contributes to the global burden of chronic and systemic diseases (e.g., cardiovascular diseases and Parkinson disease) [47,48,49,50]. NSPT, widely considered the gold standard in periodontal treatment, is not always effective unless supplemented with adjuvant agents or therapies such as ozone [51,52]. Besides being effective in treating periodontitis, ozone therapy is also applied for the resolution of other oral diseases, such as oral lichen planus [52,53].

Ozone is used as an adjunctive therapy in periodontal pocket disinfection due to its strong antimicrobial action, which stems from its oxidizing effect on microbial cell membranes [12]. It also has immunomodulatory and anti-inflammatory properties, inducing oxidative stress that activates enzymes to block inflammation and promote tissue repair and regeneration through fibroblast stimulation [14].

Its effectiveness in pain management has been demonstrated, as ozone neutralizes inflammatory mediators and accelerates wound healing via increased cell proliferation.

Ozonated olive oil is a cost-effective option, with its monounsaturated fatty acids reacting with ozone to produce compounds that enhance antimicrobial effects.

Ozonated water offers a safer alternative to gaseous ozone by reducing environmental dispersion and inhalation risk, while gaseous ozone remains advantageous for its deep tissue penetration, reaching areas inaccessible to conventional agents.

In the present review, 22 studies on ozone therapy were retrieved and analyzed qualitatively according to the type of ozone therapy used (gaseous ozone, ozonated water, or ozonated oil/gel).

Based on the results of this meta-analysis, the null hypothesis can be rejected, as a statistically significant difference (*p* < 0.0001) was observed in the variation of BOP and GI between the groups treated with ozone therapy (in all its formulations) in addition to non-surgical periodontal therapy (NSPT), compared to the groups receiving NSPT alone. Another clinical parameter that showed a favorable response was pocket depth (PPD), which also demonstrated a statistically significant reduction in the ozone-treated groups, supporting the added clinical benefit of ozone as an adjunct to conventional therapy.

In particular, half of the studies (4/8) on ozone gas evaluated the effectiveness of therapy combined with SRP. The results were discrepant, with five studies reporting statistically significant differences [27,30,31,32,33,34] between the test and the control group. In their study with a split-mouth design, Ramirez et al. [29] evaluated gaseous ozone therapy combined with SRP and SRP alone. Two endpoints were set, one at 3 weeks and one at 6 weeks.

Progression based on the GI for the teeth treated with ozone therapy suggested a favorable outcome (*p* < 0.0001), whereas no significant changes were observed in the control group. There was also a statistically significant improvement in CAL in the ozone-treated compared with the control group (*p* < 0.0001); no significant changes were recorded in the control group during treatment or at week 6.

Butera et al. [33] compared the efficacy of gaseous ozone therapy adjunctive to ultrasound treatment versus SRP combined with CHX and SRP alone. Clinical parameters were assessed at baseline (T0), 1 month (T1), 3 months (T2), and 6 months (T3). Analysis of PPD revealed a greater reduction, with improvement at 3 and 6 months in the ozone therapy group (*p* < 0.01), and a final mean reduction in PPD of 1.41 mm and 0.11 mm in the ozone group and in the SRP group, respectively. Moreover, the statistically significant improvement observed in BOP in the ozone therapy group (*p* < 0.01) provides further support for the hypothesis that ozone therapy can reduce gingival bleeding.

Uraz et al. [30] investigated the use of ozone as adjunctive therapy for chronic periodontitis and assessed its clinical, microbiological, and biochemical effects. They found that ozone treatment considerably reduced PPD, BOP, and inflammatory biomarkers in the gingival crevicular fluid. Furthermore, microbiological analysis showed a notable decrease in pathogenic bacteria following ozone therapy combined with SRP.

Skurska et al. [32] explored the impact of ozone therapy on clinical parameters and matrix metalloproteinase (MMP) levels in patients with chronic aggressive periodontitis. They observed a notable reduction in MMP levels, indicating that ozone therapy may play a role in modulating inflammation and slowing tissue degradation [34].

Similarly, Rapone et al. [26] administered gaseous ozone therapy combined with SRP and compared outcomes with SRP alone. Clinical parameters were reassessed at 3 and 6 months; at both timepoints, the ozone therapy group showed a significant reduction in PPD (*p* ≤ 0.0001). There were no statistically significant differences in mean CAL at baseline between the test and the control groups. A difference between the groups was observed at 3 months (*p* < 0.003) and continued at 6 months (*p* < 0.0001). The findings of this RCT suggest that the combined use of gaseous ozone therapy and conventional periodontal treatment may reduce the likelihood of disease progression.

Differently, other studies showed no additional benefit of ozone therapy versus standard therapy. In their RCT, Tasdemir et al. [31] found no statistically significant differences in PPD reduction in the gaseous ozone group at 3 months (*p* > 0.05). These results were shared by Dengizek et al. [28] in their full-mouth design study in which gaseous ozone therapy as an adjunct to SRP was compared with SRP + placebo (air); no statistically significant difference (*p* > 0.05) was found between the groups and the mean PPD reduction at 1 month post-treatment was 0.6 [28].

The eight studies on ozonated water reported inconsistent results. Two [40,42] found statistically significant differences (*p* < 0.001) between the test and the control group, one of which [40] reported a reduction in CAL, PI, BOP, and PPD. Both studies compared SRP alone with SRP + ozonated water. The same results for PPD were observed in a study by Ranjith et al. [36], which combined mechanical therapy + ozonated water but showed no favorable outcomes except for a decrease in PPD (*p* < 0.001). Two studies compared ozone therapy with CHX added to conventional therapy [38,39]; both reported mildly positive results and concluded that ozonated water can be considered more effective than CHX.

In their RCT with a full-mouth design, Hayakumo et al. [40] compared the efficacy of nanobubbles in irrigation water for periodontal debridement vs. a placebo (water). At 1 month and 8 months, only the experimental group (NBW3) demonstrated a statistically significant improvement at 8 weeks from baseline (*p* > 0.001). Comparison between the treatment groups revealed no statistically significant differences in BOP (%) [40].

Similarly, a split-mouth study by Al Habashneh et al. [34] investigated the efficacy of ozonated water irrigation combined with SRP vs. SRP alone vs. placebo (distilled water) in the control group. The endpoint was set at 3 months; a reduction in the PI was observed for both treatment groups; however, owing to the study’s limitations, the use of ozonated water as an adjunct to SRP did not demonstrate a statistically significant difference between the test and the control groups [34].

Five studies investigated the use of ozonated gel/oil (three using ozonated oil and two using gel), comparing ozone with CHX gel, two of which reported no positive results, while one study reported a statistically significant improvement (*p* < 0.001) with ozonated oil as monotherapy or in addition to SRP [44].

The remaining studies on oil gels found CHX gel to be more effective than oil gel. One of the studies with a split-mouth design compared the use of ozonated sunflower seed oil gel with CHX 1% gel [43]; there was a gradual increase in PPD reduction in the control group (*p* < 0.05), which continued at 6 months. In contrast, no significant differences were observed at the test site (*p* < 0.05), although an increase in reduction was noted. Significantly less reduction was noted in the test group than in the control group at 6 months (*p* < 0.05). In the other study, CHX gel therapy was found to improve CAL and GI parameters, but there were no differences between CHX gel and oil gel for the other parameters [14].

Clinical evaluation of the efficacy of ozone therapy in decreasing inflammatory indexes yielded inconsistent findings. Caution is warranted, as the lack of standardized outcome metrics (some studies measuring PPD, some GI, some BOP, and some CAL, combined or alone) does not allow a definitive conclusion to be drawn, notwithstanding the statistical evaluation using the most available comparable data.

The microbiological outcomes after ozone therapy showed it had effective antimicrobial action. Gaseous ozone demonstrated broad-spectrum action against anaerobic bacteria within a relatively short period (only 3 weeks) [31]. Longer therapy (1 to 3 months [33] reduced *P. gingivalis*, *T. forsythia*, and *P. intermedia* significantly (*p* < 0.05) and as effectively as SRP alone (control treatment). A significant reduction in almost half of pathological sites was reported as a marked improvement at 6 months [30].

Furthermore, a significant reduction in pathogens was observed at 2 months after ozonated water therapy [36], although no statistical difference was found compared with the control group (SRP + photodynamic therapy). One study [39] reported a 50% reduction in *A. actinomycetemcomitans* and a reduction in *Candida albicans* compared to the control treatment (CHX), with no effect on these bacteria.

No significant difference in the microbiological reduction in *P. gingivalis* or *T. forsythia* was reported [41] for either treatment group, although a ≥95% reduction in *P. gingivalis* was noted in the ozone group compared with only 50% in the control group. The microbiological analysis was conducted on samples collected from the subgingival area with a periodontal curette, then removal of supragingival plaque with a toothbrush and isolation of the tooth with sterile cotton rolls. The plaque was emulsified in a saline solution. The patients were classified based on the bacterial strain identified: “healthy” if only cocci or bacilli were present, “moderate risk” if bacilli predominated, and “high risk” if spirochetes or other motile microorganisms were identified. A decrease in bacterial load was noted in the ozone-treated group between weeks 0 and 3 (*p* < 0.0001), whereas no significant difference in the control group was observed throughout the study [41]. Furthermore, other studies [41,42] observed no differences between groups despite clinical improvements.

In their evaluation of the efficacy of ozonated olive oil as adjunctive therapy, Patel et al. [44] collected subgingival plaque samples from two sites in each quadrant at baseline, 4 weeks, and 8 weeks for analysis of total bacterial count and frequency of eight presumed periodontal pathogens via polymerase chain reaction (PCR). No significant differences were found between the groups at T0. The use of adjunctive ozone therapy in combination with SRP led to a significant improvement (*p* < 0.001) in microbiological parameters over time compared with the control groups. A significant improvement (*p* < 0.001) in microbiological parameters over time with no documented side effects was reported for ozone monotherapy [44]. Lastly, Gandhi et al. [45] collected subgingival plaque from within the periodontal pocket using a sterile curette. Ozone therapy led to a statistically significant reduction in the levels of *P. gingivalis* and *A. actinomycetemcomitans* (*p* < 0.05) [45].

Despite discordant results, it can be stated that ozone, especially in gaseous or water form, seems to possess antimicrobial potential against *A. actinomycetemcomitans*, *P. gingivalis, T. forsythia*, and *Candida albicans*. Many studies did not specify the bacterial strain(s) but rather generally referred to subgingival plaque; this lack of detail limits the rationale for adequate comparison. Another limitation for the proper analysis of outcomes is the absence of microbiological data analysis at long-term follow-up (more than 3 months). As variability in outcomes can be related to methodology and study design, concentration of agents, type of pathogens analyzed, and exposure time, more standardized studies are needed to clarify the microbiological efficacy of ozone in periodontology.

Concerning the heterogeneity of outcomes, it can be stated that ozone, especially in gaseous and water form, can reduce inflammation as measured by the clinical parameters BOP and the GI, which are the primary indexes that can be easily assessed in daily practice after treatment.

This study shows considerable variability in the methodology used to administer ozone, with variations in concentration, frequency, and exposure time. This increases the heterogeneity of the study, thus representing a significant limitation, as it could affect the validity of the conclusions drawn.

A further limitation of this study is the characteristics of the patients, such as their initial oral hygiene status and smoking habits, which could potentially influence the results of non-surgical periodontal therapy.

Improvement can be followed by changes or stability in indexes of periodontal healing in long-term follow-up (decreased PPD, increased CAL), which are desirable for stabilizing pocket depth after NSPT. Moreover, immune response modulation involves microbiological balance, an important parameter which is less easy to assess in daily practice but essential for evaluating outcome stability after therapy.

As regards patient-reported long-term side effects, ozone can be safely administered in different forms, except by inhalation, which can damage the lungs. While ozone has many good uses, it can be harmful if too much is inhaled, which is more of a risk for clinicians than for patients [53]. Inhalation of too much ozone can cause headaches, vomiting, and throat irritation. Furthermore, ozone treatment should not be administered in patients with a history of heart attack, thyroid conditions, alcohol poisoning, severe anemia, low platelet count, bleeding, or pregnancy. It can be concluded that, when used following guidelines, ozone therapy can be considered generally safe with no side effects.

Finally, notwithstanding its potential benefits, ozone therapy carries a high cost like other supplementary treatments such as laser therapy and advanced topical agents. The extra expense often limits its availability, particularly for patients without comprehensive health insurance coverage. To address this limitation, the adoption of ozone therapy by university dental clinics can offer a possible solution. University settings usually have access to research funding, state-of-the-art equipment, and a pool of skilled clinicians and students eager to gain hands-on experience. By offering ozone therapy in an academic environment, its cost can be reduced through shared resources and the use of treatment as a teaching tool. Additionally, university clinics often operate on a sliding scale fee structure, which makes treatment more affordable for a broader range of patients.

## 5. Conclusions

The present systematic review reports that ozone has been extensively tested as a therapy adjunctive to conventional mechanical instrumentation, with only one study testing ozone in monotherapy. Owing to the heterogeneity of the study results, no definitive conclusions can be drawn on whether one type of ozone is superior to another, nor that the use of ozone can be always considered an effective adjunctive therapy. What can be stated is that ozone may help in the treatment of periodontal disease as an aid to standard therapy, which relies on the use of pocket instrumentation as the first-line standard approach.

Given the heterogeneity of the included studies and the methodological limitations observed, future research should focus on well-designed randomized controlled trials with larger samples, longer follow-up periods, and clearly defined ozone application protocols. Standardization of clinical outcome measures, such as probing depth, bleeding index, and microbiological markers, is strongly recommended. Furthermore, future studies should investigate the efficacy of ozone as an adjunctive treatment in specific patient subgroups, such as those with advanced periodontitis or systemic conditions such as diabetes.

These elements would contribute to exploring the use of ozone in non-surgical periodontal therapies and to the development of evidence-based clinical guidelines.

## Figures and Tables

**Table 1:** Inclusion and exclusion criteria.

Inclusion Criteria	Exclusion Criteria
Adult patients diagnosed with periodontitis;	Patients with systemic disease;
Application of ozone (gas, liquid, gel, oily formulation);	Implantology and periodontal surgery;
Bleeding on probing as the primary clinical parameter;	Patients < 18 years of age;
Non-surgical periodontal therapy	Reported no diagnosis of periodontitis;
	Were in vitro or animal studies;
	The topic was outside the scope of the present review

**Table 2:** RCTs on gaseous ozone therapy.

Authors	Year	Study Design	Country	Sample Size	Age/Test Age/Control	Sex	Test and Control Interventions (Ozone Device; Ozone Dose; Application Frequency)	Follow-Up	Outcome
Yilmaz et al. [27]	2013	split-mouth	Turkey	30	1. 41.4 ± 8.86 2. 43.0 ± 5.01 3. 41.4 ± 4.62	F 18 M 12	1. SRP + gas (OzonytronX; NI; twice a week (2 wks) after SRP) 2. SRP + Er:YAG laser 3. SRP	Baseline 3 mths	PI, SBI, RAL
Butera et al. [33]	2021	split-mouth	Italy	48	42.16	F 20 M 28	1. US + gas (NI) 2. SRP + CHX gel 0.20% 3. SRP	Baseline 1 wk, 1 mth, 3 mths, 6 mths	BOP, BP, PS, CAL, PI, microbiological analysis
Dengizek et al. [28]	2019	full-mouth	Turkey	40	42.4 ± 6.7	F 16 M 21	1. SRP + gas (Ozone DTA; NI; twice on day 3 and 8 after SRP) 2. SRP + placebo	Baseline, 1 mth	PI, GI, PD, CAL, TAS, NO, 8-OHdG, MDA, TGF-β
Ramirez Peña et al. [29]	2022	split-mouth	Spain	32	34–64	F 22 M 10	1. SRP + gas (Ozoneline^®^; 2 mL of ozone 30 μg/mL; once a week (4 wks) after SRP) 2. SRP + air	Baseline, 3 wks, 6 wks	GI, CAL, MOV, VAS, Microorganisms qualitatively compared
Tasdemir et al. [31]	2019	split-mouth	Turkey	36	43.7	F 18 M 18	1. SRP + gas (OzonytronX; 75 μg/mL; twice a week (2 wks) after SRP) 2. SRP + placebo	Baseline, 3 mths	PI, GI, PD, BOP%, CAL, PD% > 5 mm and CAL % ≥ 3 mm), IL-1β, Hs-CRP
Rapone et al. [26]	2022	full-mouth	Italy	90	1. 51.62 ± 9.56 2. 49.88 ± 10.54	F 16 M 74	1. SRP + gas (Ozone DTA; NI; 1/2 cycles after SRP) 2. SRP	Baseline, 3 mths, 6 mths	BOP, PPD, GM, CAL
Skurska et al. [32]	2010	split-mouth	Poland	52	25–68	F 35 M 17	1. SRP 2. SRP + gas (OzonyMed^®^; 42.2 μg/Ozone ml of oxygen; 3 times (every second day for 1 wk) 3. SRP + gas	Baseline, 2 wks, 2 mths	PI, API, BOP, SBI, PPD, CAL
Uraz et al. [30]	2018	split-mouth	Turkey	18	40 ± 6.51	F 9 M 9	1. SRP + gas (Ozone DTA; 2100 ppm with 80% oxygen; 3 times every third day for 1 wk) 2. SRP	Baseline 1 wk, 1 mth, 3 mths	PI, GI, BOP, PPD, Microorganisms qualitatively compared

Abbreviations: wk(s) denotes week(s), mth(s) month/s, NI no information, RCT randomized controlled trial, SRP scaling and root planing, PI plaque index, GI gingival index, PPD probing pocket depth, CAL clinical attachment loss, BOP bleeding on probing, SBI sulcus bleeding index, 8-OHdG 8-hydroxyguanosine, MDA malondialdehyde, TGF-β transforming growth factor beta, Hs-CRP high-sensitivity C-reactive protein, IL-1β interleukin-1, US ultrasound.

**Table 3:** RCTs on ozonated water.

Authors	Year	Study Design	Country	Sample Size	Age/Test Age/Control	Sex	Test and Control Interventions (Ozone Device; Ozone Dose; Application Frequency)	Follow-Up	Outcome
Shrivastava et al. [41]	2022	full-mouth	India	30	30–65	/	1. SRP + ozonated water (NI; 20-gauge blunt syringe for 30/45 s once after SRP) 2. SRP + distilled water (placebo)	Baseline, 14 days, 21 days, 2 mths	PI, GI, PPD, microbiological analysis
Mehrotra et al. [35]	2023	split-mouth	India	26	30–65	F 12 M 14	1. SRP + ozonated water (NI; 22-gauge syringe for 5/10 min once a mth (2 mths) after SRP) 2. SRP + PDT	Baseline, 1 mth 2 mths	PI, GI, PPD, CAL, microbiological analysis
Alsakr et al. [39]	2023	split-mouth	Arabia	46	44.41 ± 9.1	F 15 M 31	1. SRP + ozonated water (NI; 5/20 μg/mL once after SRP) 2. SRP	Baseline, 6 wks	PD, CAL, PI, BOP
Vasthavi et al. [42]	2020	full-mouth	India	24	30–65	/	1. SRP + ozonated water (NI; 20-gauge blunt syringe for 30/45 s once after SRP) 2. SRP + distilled water (placebo)	Baseline, 14 days, 21 days, 2 mths	PI, GI, PPD, microbiological analysis
Ranjith et al. [36]	2022	split-mouth	India	50	1. 49.23 ± 8.87 2. 47.55 ± 9.69	F 18 M 24	1. SRP + ozonated water (Ozone Generator ADC; 2 mL per tooth for 1 min once after SRP) 2. SRP + saline irrigation (placebo)	Baseline, 4 wks	FMPS, FMBS, PPD, CAL, IL-1β
Al Habashneh et al. [34]	2015	split-mouth	Jordan	41	1. 39 ± 13.7 2. 39 ± 10.2	F 28 M 13	1. SRP + ozonated water (Hyper nedezon Comfort; 75/85 μg/mL once after SRP) 2. SRP + ozonated water (placebo)	Baseline, 3 mths	PI, GI, BOP, PPD, REC, CAL, CRP
Hayakumo et al. [40]	2013	full-mouth	Japan	22	1. 45.90 13.8 2. 45.9 14.8	F 6 M 16	1. FMMD + NBW3 (nanobubble ozone water irrigation) (Nanobubble Generating Technology; NI; once after SRP) 2. FMMD + tap water (placebo)	Baseline, 1 mth, 2 mths	PPD, CAL, BOP%, microbiological analysis
Kshitish et al. [38]	2010	split-mouth	India	17	20–60	/	1. SRP + ozonated water (Kent Ozone Dental Jet; different speeds and pressures (350/500 kPa) 20-gauge blunt syringe for 5/10 min once after SRP) 2. SRP + gel CHX 0.20%	Baseline, 1 wk, day 8 to 18	PI, GI, GBI, microbiological analysis
Kaur et al. [37]	2019	split-mouth	India	20	30–60	/	1. SRP + Ozonated water (Kent Ozone Dental Jet; NI; once after SRP) 2. SRP + CHX 0.2%	Baseline 4 wks, 3 mths	GI, PPD, CAL

Abbreviations: wk(s) denotes week(s), mth(s) month/(s); NI No Information, RCT randomized controlled trial, SRP scaling and root planing, PI plaque index, GI gingival index, PPD probing pocket depth, CAL clinical attachment loss, BOP bleeding on probing, SBI sulcus bleeding index, FMPS Full-Mouth Plaque Score; FMBS Full-Mouth Bleeding Score; GBI gingival bleeding index.

**Table 4:** RCTs on ozonated olive oil/gel.

Authors	Year	Study Design	Country	Sample Size	Age/Test Age/Control	Sex	Test and Control Interventions (Ozone Product Name; Ozone Dose; Application Frequency)	Follow-Up	Outcome
Nambiar et al. [46]	2022	split-mouth	India	30	38.2	/	1. SRP + ozonated olive oil gel (pur03 LLC; NI; 1 wk after SRP) 2. SRP + CHX gel	Baseline, 3 mths	PPD, SBI
Gandhi et al. [45]	2019		India	25	30–60	/	1. SRP + ozonated olive oil (NI; 2 mL with a 28-gauge needle after SRP and 2 wks later) 2. SRP + CHX 0.20%	Baseline, 3 mths	PI, GI, PPD, CAL, microbiological analysis
Colombo et al. [14]	2021	split-mouth	Italy	10	50	F 6 M 4	1. SRP + ozonated bio-olive oil gel (GeliO3; NI; once after SRP) 2. SRP + CHX gel 1%	Baseline, 1 mth, 3 mths	PPD, CAL, GI, BOP, PI
Scribante et al. [43]	2024	split-mouth	Italy	30	49.4 ± 9.32	F 15 M 15	1. NMPD + Ozoral gel + Ozoral Pro (ozonized sunflower seed oil) (Ozoral gel + Ozoral Pro; NI; Ozoral gel in single daily application for 1 wk (at home) and Ozoral Pro: after SRP (in office)) 2. NMPD + CHX gel 1%	Baseline, 1 mth, 2 mths, 6 mths	CAL, PPD, BOP
Patel et al. [44]	2012	split-mouth	India	20	42.64 ± 7.8	F 8 M 12	1. SRP + ozonated olive oil gel (NI; 140 mg/mL after SRP at baseline and after 2, 4, and 6 wks) 2. ozonated olive oil gel monotherapy 3. SRP + CHX gel 1% 4. SRP	Baseline,2 wks, 4 wks, 6 wks, 8 wks	VAS, PI, GI, SBI, PPD, CAL

Abbreviations: wk(s) denotes week(s), mth(s) month/(s); NI No information, RCT randomized controlled trial, SRP scaling and root planing, PI plaque index, GI gingival index, PPD probing pocket depth, CAL clinical attachment loss, BOP bleeding on probing, SBI sulcus bleeding index, VAS visual analogue scale, CHX chlorhexidine.

**Table 5:** Assessment of quality of evidence for ozone therapy.

Outcomes	Anticipated Absolute Effects * (95% CI)		Relative Effect (95% CI)	No. of Participants (Studies)	Certainty of the Evidence (GRADE)	Comments
	Risk with Control	Risk with Ozone in Adjunctive Therapy				
BOP. Follow-up: 1–3 months	Mean reduction 41.76%	Mean 6.73% lower (9.11 to 4.34 lower)	-	297 (7 RCTs)	⨁⨁⨁◯ Moderate ^a,b,c,d^	Ozone in adjunctive therapy reduces bleeding on probing.
GI scale 0 to 3; follow-up: 1–3 months	Mean reduction 0.72	Mean 0.32 lower (0.41 to 0.24 lower)	-	301 (6 RCTs)	⨁⨁⨁◯ Moderate ^a,b,c,d^	Ozone in adjunctive therapy may reduce gingival index.
PPD reduction with ozone gas; follow-up 1–3 months	Mean reduction 1.36 mm	Mean 0.28 mm lower (0.42 to 0.12 lower)	-	219 (5 RCTs)	⨁⨁⨁◯ Moderate ^a,b,c,d^	Ozone gas in adjunctive therapy may slightly reduce PPD.
PPD reduction with ozone water; follow-up 1–3 months	Mean reduction 1.27 mm	Mean 0.22 mm lower (0.37 to 0.08 lower)	-	208 (5 RCTs)	⨁⨁⨁◯ Moderate ^a,b,c,d^	Ozone water in adjunctive therapy may slightly reduce PPD.
PPD reduction with ozone; follow-up 1–3 months	Mean reduction 1.31 mm	Mean 0.25 mm lower (0.35 to 0.15 moderate)	-	427 (10 RCTs)	⨁⨁⨁◯ Moderate ^a,b,c,d^	Ozone in adjunctive therapy may slightly reduce PPD.
CAL reduction after ozone vs. SRP; follow-up 1–3 months	Mean reduction 1.63 mm	Mean 0.4 mm lower (0.49 to 0.31 lower)	-	344 (6 RCTs)	⨁⨁⨁◯ Moderate ^a,b,c,d^	Ozone in adjunctive therapy may slightly reduce CAL.

Notes. (a) Visual inconsistency and statistical analysis also showing heterogeneity; (b) the evidence directly answers the health care question; (c) RTC; (d) continuous outcome; BOP denotes bleeding on probing, PPD probing pocket depth; GI gingival index, CAL clinical attachment loss, SRP scaling and root planing. * The risk in the intervention group (and its 95% CI) is based on the assumed risk in the comparison group and the relative effect of the intervention (and its 95% CI). CI denotes confidence interval, BOP bleeding on probing.

**Table 6:** Assessment of quality of evidence for ozone gel.

Outcomes	Anticipated Absolute Effects * (95% CI)		Relative Effect (95% CI)	Sample Size (No of Studies)	Certainty of Evidence (GRADE)	Comments
	Risk with Chlorhexidine in Adjunctive Therapy	Risk with Ozone Gel in Adjunctive Therapy				
GI at mean follow-up of 3 months	Mean reduction 1.16 mm	Mean 0.1 mm (0.2 to 0.01 mm lower)	-	110 (3 RCTs)	⨁⨁⨁ Moderate ^a,b,c^	Little to no difference in GI reduction with ozone gel in adjunctive therapy
PPD at mean follow-up of 3 months	Mean reduction 1.03 mm	Mean 0.68 mm (0.81 to 0.54 lower)	-	300 (5 RCTs)	⨁⨁⨁⨁ High ^a,b,c^	Ozone gel in adjunctive therapy may reduce PPD

Notes * Risk in the intervention group (and its 95% confidence interval) is based on the assumed risk in the comparison group and the relative effect of the intervention (and its 95% CI); (a) wide confidence interval likely due to heterogeneity; (b) RTC; (c) continuous outcome; CI denotes confidence interval, PPD probing pocket depth, GI gingival index.

## Data Availability

Data are available from the corresponding authors upon reasonable request.
